# Towards a multi protein and mRNA expression of biological predictive and distinguish model for post stroke depression

**DOI:** 10.18632/oncotarget.11105

**Published:** 2016-08-05

**Authors:** Yingying Yue, Haitang Jiang, Rui Liu, Yingying Yin, Yuqun Zhang, Jinfeng Liang, Shenghua Li, Jun Wang, Jianxin Lu, Deqin Geng, Aiqin Wu, Yonggui Yuan

**Affiliations:** ^1^ Department of Psychosomatics and Psychiatry, ZhongDa Hospital, School of Medicine, Southeast University, Nanjing, PR China; ^2^ Institute of Psychosomatics, Medical School of Southeast University, Nanjing, PR China; ^3^ School of Information Science and Engineering Southeast University, Nanjing, PR China; ^4^ Department of Neurology, Jiangning Nanjing Hospital, Nanjing, PR China; ^5^ Department of Neurology, The Affiliated First Hospital of Nanjing Medical University, Nanjing, PR China; ^6^ Department of Neurology, The Peoples’ Hospital of Gaochun County, Nanjing, PR China; ^7^ Department of Neurology, Affiliated Hospital of Xuzhou Medical College, Xuzhou, PR China; ^8^ Department of Psychosomatics, The Affiliated First Hospital of Suzhou University, Suzhou, PR China

**Keywords:** post stroke depression, neurotrophic factors, protein, mRNA, Pathology Section

## Abstract

Previous studies suggest that neurotrophic factors participate in the development of stroke and depression. So we investigated the utility of these biomarkers as predictive and distinguish model for post stroke depression (PSD). 159 individuals including PSD, stroke without depression (Non-PSD), major depressive disorder (MDD) and normal control groups were recruited and examined the protein and mRNA expression levels of vascular endothelial growth factor (VEGF), vascular endothelial growth factor receptors (VEGFR2), placental growth factor (PIGF), insulin-like growth factor (IGF-1) and insulin-like growth factor receptors (IGF-1R). The chi-square test was used to evaluate categorical variable, while nonparametric test and one-way analysis of variance were applied to continuous variables of general characteristics, clinical and biological changes. In order to explore the predictive and distinguish role of these factors in PSD, discriminant analysis and receiver operating characteristic curve were calculated. The four groups had statistical differences in these neurotrophic factors (all *P* < 0.05) except VEGF concentration and IGF-1R mRNA (*P* = 0.776, *P* = 0.102 respectively). We identified these mRNA expression and protein analytes with general predictive performance for PSD and Non-PSD groups [area under the curve (AUC): 0.805, 95% CI, 0.704-0.907, *P* < 0.001]. Importantly, there is an excellent predictive performance (AUC: 0.984, 95% CI, 0.964-1.000, *P* < 0.001) to differentiate PSD patients from MDD patients. This was the first study to explore the changes of neurotrophic factors family in PSD patients, the results intriguingly demonstrated that the combination of protein and mRNA expression of biological factors could use as a predictive and discriminant model for PSD.

## INTRODUCTION

Stroke is a major disease of high morbidity and mortality, 2.5 millions new stroke cases increasing each year in china [1]. Post stroke depression (PSD) is a frequent complication with approximately 30% prevalence that worsens rehabilitation outcomes and confers substantial risk for suicide [2–4]. Moreover, PSD in turn increased the risk of stroke recurrence [5]. The pathogenesis of PSD as well as the differentiation from major depressive disorder (MDD) remains unknown.

It is widely known that the etiology is heterogenous and complex involved in the development of PSD, including psychological and biological mechanisms. Stroke as an acute excessive stress for a prolonged period is enormously harmful to both mental and physical conditions, then accelerate the occurrence of PSD [6]. These disorders may be resulting from decreased neurogenesis and neuronal plasticity. A group of trophic and growth factors including vascular endothelial growth factor (VEGF), placental growth factor (PIGF), and insulin-like growth factor (IGF) may contribute to alleviating emotional or psychological stresses. In addition, recent studies have indicated that impairment in neurovascular dysfunction may be an important factor in the pathogenesis of ischemic stroke, depression, Alzheimer's disease and multiple sclerosis [7, 8].

Previous studies discussed the role of VEGF in the development and treatment of the stroke and depression [9–11]. VEGF was the founding member in VEGF family and initially isolated as a factor increasing vascular permeability [12]. Further study found that it could regulate vessel and neuronal wiring, and then play roles in neurogenesis, synaptic plasticity and neuroprotection [8]. The expression of VEGF is tightly regulated by (inflammatory) cytokines and proteins, including interleukin-1 (IL-1), IL-6, IGF-1 and hypoxia which is the key factors [13]. In the nervous system, VEGF signaling activity depends on VEGF receptors (VEGFR2, termed kinase insert-domain containing receptor, KDR in humans or Flk1 in mice), which is the best characterized signaling receptor, stimulates migration, proliferation, and survival of various neural cell types [14]. In addition, VEGF/KDR signal mediates anti-depressive like effect [15]. In ischemia stroke, autopsy and histological studies showed that hypoxia-inducible factor triggered many downstream molecules including the expression of VEGF and its receptors, which could drive angiogenesis in the ischemic penumbra [16, 17]. In depressive patients, some researchers also found that serum protein concentration and mRNA expression level of VEGF and VEGFR2 were higher than healthy individuals [11, 18, 19]. However, the results were not consistent in clinical data of depression [20]. PIGF is another factor of VEGF family, which stimulates angiogenesis through forming VEGF/PlGF heterodimers and thereby amplifies VEGF-driven signaling [21].

IGF-1, the only neurotrophic factor that may be regulated by the immune system, has gained great attention in diseases affecting the central nervous system (e.g. depression and stroke) on account of enhancing neuroprotective effects, neuronal survival and plasticity in the brain [22]. Lee et al [23] demonstrated that the mRNA of IGF-1 and IGF-1 receptor (IGF-1R) differed with time dependent in maternal separation stressed animals. In the light of the discovery of alterations in IGF-1 levels in depression-like states, its antidepressant-like activity was discovered in various animal models of depression [24, 25]. Human studies conducted in different laboratories showed discrepancy finding in the peripheral blood levels in depressed patients [26, 27]. However, the changes of the IGF-I axis in PSD are still lack empirical evidence, despite accumulating knowledge have been gained about it.

Taking into account the prominent role of neurotrophic factor family in both cerebrovascular disease and depression, they could be molecular links in PSD disease. So we hypothesize that peripheral biological factors are related to the development of PSD and they may serve as predictive and discriminant indicators for PSD.

## RESULTS

### Demographic and neuropsychological results

The demographic and neuropsychological characteristics are summarized in Table 2. There were no differences in education level among the four groups (*P* = 0.305), nevertheless others had significant differences including age, gender, Hamilton depression rating scale (HDRS) and mini mental state examination (MMSE) (all *P* < 0.05).

**Table 1:** The primers for qPCR

Gene name	Primer	Sequence
VEGF	VEGF-F	TGCTTCTGAGTTGCCCAGGA
	VEGF-R	TGGTTTCAATGGTGTGAGGACATAG
VEGFR2	VEGFR2-F	CGGCAAATGTGTCAGCTTTG
	VEGFR2-R	CACGTGGAAGGAGATCACCC
PIGF	PIGF-F	GCACCACTGATAGAGTTGGCA
	PIGF-R	GCTTTGAGGTTTGGTCCTAACA
IGF-I	IGF-I-F	GCATCTCTTCTACCTGGCGC
	IGF-I-R	AAAGCCCCTGTCTCCACACA
IGF-1R	IGF-1R-F	GGCATACCTCAACGCCAATA
	IGF-1R-R	CAGCCCTTTCCCTCCTTT
GAPDH	GAPDH-F	AGCCACATCGCTCAGACAC
	GAPDH-R	GCCCAATACGACCAAATCC

Note: qRT-PCR: quantitative real-time polymerase chain reaction; VEGF: vascular endothelial growth factor; VEGFR2: vascular endothelial growth factor receptor 2; PIGF: Placental growth factor; IGF-1: insulin-like growth factor; IGF-1R: insulin-like growth factor receptor; GAPDH: glyceraldehyde-3-phosphate dehydrogenase.

**Table 2:** Demographic and neuropsychological data among four groups

Item	PSD group (n=39)	Non-PSD group (n=42)	MDD group (n=40)	NC group (n=38)	F/X^2^/Z	*P* value
Age (years)	62.44±10.34	61.10±6.58	58.70±9.92	57.58±5.28	2.761	0.044^a^
Gender (male/female)	20/19	25/17	9/31	22/16	14.316	0.003^b^
Education level (years)	8.15±4.36	9.43±4.01	9.40±4.77	8.24±2.56	1.218	0.305^a^
Active smokers n (%)	16(41.0%)	21 (50.0%)	9 (22.5%)	-	6.796	0.033^b^
Alcohol consumption n (%)	9(23.1%)	16(38.1%)	16 (40.0%)	-	3.034	0.219
HDRS	16.00±5.45	3.36±2.02	19.45±4.75	2.18±1.98	203.322	<0.001^a^
MMSE	22.64±6.80	27.00±2.45	27.08±1.45	28.47±1.45	28.46	<0.001^c^
NIHSS	5.64±4.69	2.64±2.90	-	-	−3.435	0.001^d^
mRS	2.92±1.24	1.74±1.11	-	-	−4.084	<0.001^d^
BI	56.67±30.16	83.33±22.92	-	-	−3.991	<0.001^d^

Note: Data reported as mean ± SD and ratio. PSD: post stroke depression; Non-PSD: stroke without depression; MDD: major depressive disorder; NC: normal control. HDRS: Hamilton depression rating scale; MMSE: mini mental state examination. NIHSS: National Institutes of Health Stroke Scale, mRS: modifed Rankin Scale, BI: Barthel Index.

a one-way ANOVA.

b Chi square test.

c Kruskal-Wallis test.

d Mann-Whitney U test.

### The protein and mRNA expression level

The four groups had statistical differences in these five factors (*P* < 0.05) except VEGF concentration and IGF-1R mRNA (X^2^_Kruskal-Wallis, df(3)_ = 1.105, *P* = 0.776; X^2^_Kruskal-Wallis, df(3)_ = 6.197, *P* = 0.102 respectively). Compared with normal controls (NC), there was decreased VEGFR2 content in PSD (F_Bonferroni_ = −489.29, standard error (SE) = 68.44, *P* < 0.05, 95%confidence interval (CI), −672.21~-306.37), stroke without depression (Non-PSD) (F_Bonferroni_ = −373.62, SE = 67.22, *P* < 0.05, 95%CI, −553.29~-193.95) and MDD group (F_Bonferroni_ = −423.01, SE = 68.44, *P* < 0.05, 95%CI, −605.93~-240.09), but increased PIGF protein in PSD (F_Dunnett T3_ = 6.48, SE = 0.96, *P* < 0.05, 95%CI, 3.92~9.04) and Non-PSD groups (F_Dunnett T3_ = 3.93, SE = 0.94, *P* < 0.05, 95%CI, 1.42~6.44). Moreover, the IGF-1 mRNA level was lower in MDD than NC patients (post hoc nonparametric test, *P* < 0.05). In contrast with MDD group, PSD (F_Dunnett T3_ = 7.88, SE = 0.95, *P* < 0.05, 95%CI, 5.35~10.41) and Non-PSD (F_Dunnett T3_ = 5.33, SE = 0.93, *P* < 0.05, 95%CI, 2.85~7.81) groups had raised PIGF protein, decreased VEGF, VEGFR2, PIGF and IGF-1 in mRNA field by post hoc nonparametric test (all *P* < 0.05). While it was displayed reduced IGF-1 in Non-PSD group (F_Bonferroni_ = −26.98, SE = 9.71, *P* = 0.037, 95%CI, −52.93~-1.02) and elevated IGF-1R in PSD of protein (post hoc nonparametric test, *P* < 0.05) aspect related to MDD patients (see Table 3).

**Table 3:** The results of neurotrophic factors in four groups

	PSD group (n=39)	Non-PSD group (n=42)	MDD group (n=40)	NC group (n=38)	F/H/X^2^	*P* value
**Protein level**						
VEGF(pg/ml)^‡^	328.24±212.55	316.63±221.50	288.82±193.95	350.09±244.38	1.105	0.776^c^
VEGFR2(pg/ml)^§^	1160.34±249.34^#^	1276.01±300.12^#^	1226.62±231.14^#^	1649.63±395.83	17.904	<0.001^a^
PIGF(pg/ml)	16.55±5.21*^#^	14.00±5.19*^#^	8.67±3.01	10.06±2.56	23.424	<0.001^a^
IGF-l(ng/ml)	113.68±51.46	109.62±34.54*	136.60±39.02	124.29±49.48	3.429	0.019^a^
IGF-lRCpg/ml)^¶^	243.32±146.69*	171.16±50.85	162.40±49.43	169.38±58.68	9.503	0.023^c^
**mRNA relative expression level**						
VEGF^‡^	0.44±0.36*	0.45±0.34*	0.21±0.19	1.00±1.60	15.364	0.002^c^
VEGFR2**	0.44±0.41*	0.42±0.37*	0.14±0.12	1.00±1.71	27.519	<0.001^c^
PIGF^††^	0.35±0.35*	0.36±0.36*	0.13±0.10	1.00±1.67	15.025	0.002^c^
IGF-1^‡‡^	0.50±0.39*	0.63±0.62*	0.11±0.08^#^	1.00±1.69	39.974	<0.001^c^
IGF-1R	0.68±0.60	0.55±0.30	0.66±0.38	1.00±0.75	6.197	0.102^c^

Note:

a one-way ANCOVA, gender and age as covariant.

c Kruskal-Wallis test.

# *P* < 0.05 compared with HC group.

* *P* < 0.05 compared with MDD group.

‡ two abnormal data were excluded (one PSD and one MDD patients on protein level, one Non-PSD and one MDD patients on mRNA level);

§ one abnormal data in MDD group were excluded;

¶ six abnormal data were excluded (one PSD patients, two Non-PSD patients, one MDD patients, two NC subject);

** four abnormal data were excluded (one outlier in each group);

†† three abnormal data were excluded (one outlier in PSD, Non-PSD and MDD respectively);

‡‡ eleven abnormal data were excluded (three PSD patients, two Non-PSD patients, four MDD patients, two NC subject).

It was noted that the mRNA transcriptional of IGF-1R was undetermined in 33 participants consisting of 12 PSD, 11 Non-PSD and 10 NC individuals, notwithstanding we tried our best to carry preliminary experiment many times by designing several pairs of specific qPCR primers, changing the experimental conditions, etc.

### The predictive function of neurotrophic factors in PSD and Non-PSD patients

The receiver operating characteristic (ROC) analysis showed that the area under the curve (AUC) of mRNA and protein of multi-factors different among four groups as well as the combination of these two parts were 0.619 (SE = 0.067, 95% CI, 0.488-0.749, *P* = 0.081), 0.779 (SE = 0.056, 95% CI, 0.670-0.888, *P* < 0.001) and 0.805 (SE = 0.052, 95% CI, 0.704-0.907, *P* < 0.001) respectively in PSD and Non-PSD groups.

### The distinguish function of neurotrophic factors in PSD and MDD patients

**Figure 1:**
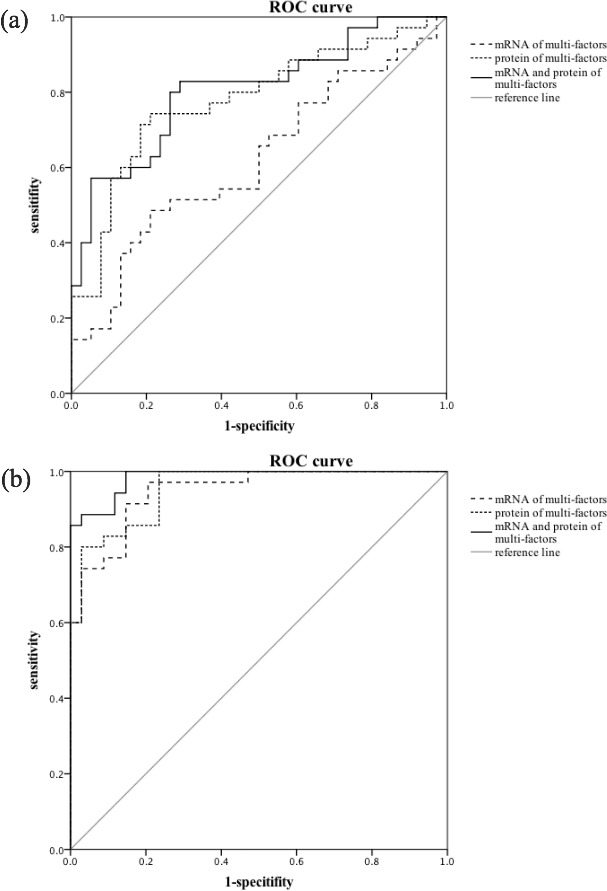
ROC curves and predictive performance **a.** The AUC of mRNA and protein of multi-factors different among four groups as well as the combination of these two parts were 0.619 (95% CI, 0.488-0.749, *P* = 0.081), 0.779 (95% CI, 0.670-0.888, *P* < 0.001) and 0.805 (95% CI, 0.704-0.907, *P* < 0.001) respectively established PSD and Non-PSD patients. **b.** In PSD and MDD patients, the mRNA of multi-factors, the protein of multi-factors and the combination of these two parts could serve as excellent distinguishing models with the AUC of 0.947 (95% CI, 0.900-0.994, *P* < 0.001), 0.954 (95% CI, 0.911-0.996, *P* < 0.001) and 0.984 (95% CI, 0.964-1.000, *P* < 0.001).

What is more meaningful was that the mRNA and protein of multi-factors different among four groups as well as the combination of these two parts could serve as excellent distinguishing models with the AUC of 0.947 (SE = 0.024, 95% CI, 0.900~0.994, *P* < 0.001), 0.954 (SE = 0.022, 95% CI, 0.911~0.996, *P* < 0.001) and 0.984 (SE = 0.010, 95% CI, 0.964~1.000, *P* < 0.001) revealed in Figure 1.

## DISCUSSION

The identification of peripheral markers of depression would be particularly useful for attempting to clarify the pathogenic mechanism of this multifactorial complex disorder and to identify discriminate PSD from MDD. Consistent evidence from biochemical studies in depressed patients reported alterations in peripheral levels of the neurotrophic factors of cell survival and plasticity [28, 29]. In this work, we found that VEGF transcriptional level, IGF-1R serum concentration, both the protein and mRNA level of VEGFR2, PIGF, IGF-1 have significant differences among different diseases. In addition, we surprisingly demonstrated for the first time the potential of a biomarker model to provide blood-based predictive and differentiated indicators for PSD.

In addition to the classical brain derived neurotrophic factor (BDNF), VEGF is another promising molecular involved in neurogenesis and synaptic plasticity. It is a pity that there is no significant difference of VEGF protein among four groups in the present study. Our negative findings were in partly accordance with a study reporting no alteration in VEGF serum level at baseline and after treatment in MDD patients [30]. Nevertheless, in the gene transcriptional level, the VEGF and VEGFR2 were lower in MDD than PSD and Non-PSD group. Above findings suggest the mRNA expression level were all reduced in morbid state, but decline degree are different in various diseases. When considering the reason of stress, which can induce or exacerbate depression, it was reasonable to comprehend less VEGF expression both in the hippocampus and periphery level [31]. Low VEGF/VEGFR2 weakened the neurogenesis and synaptic plasticity, then further induced the occurrence and progression of depression. Our results evidenced the theory of “vascular niche hypothesis of adult neurogenesis” which proposed the need of vascular recruitment associated to active sites of neurogenesis formed by proliferation [32]. The anatomical parallelisms between vessel and nerve patterning were the basis of the function of vascular permeability, mitogen, angiogenic actions, proliferation, migration and survival of nerve cells of different types [33]. The down-regulation of VEGF and receptors weakened the protective and support effect to the vessels and nerves, which susceptibility to depression. In contrast to the action of stress, expression studies in animal models identified increased VEGF expression and the signaling through the Flk-1 are required for the induction of neurogenesis and antidepressant effect operated by pharmacological and other antidepressant therapies (e.g. electroconvulsive seizures) [15, 34].

The role of its homolog, PlGF, was increased in protein and mRNA expression aberrant in PSD and Non-PSD patients compared with MDD group. It is often used in the studies of other diseases such as pregnancy complicated by hypertensive disorders, pulmonary hypertension and preeclampsia [35–37]. Previous study demonstrated that it contributed to angiogenesis and plasma extravasation in ischemia and other pathological conditions [21]. But it received little attention in depressive disorders yet. The present study displayed the PIGF was increased in PSD and Non-PSD groups, the possible explanations were as follows. First of all, the augmentation of PlGF protein detected under post stroke conditions might be due to post-transcription regulation. In the second place, the feasible reason was an improved stability of structure of the protein under hypoxia in stroke survivors regardless of depression [38]. Thirdly, the mechanism of PIGF involved in depression may synergize with VEGF, binding to its receptor (Flt1) lead to inter-molecular crosstalk with Flk1, which amplifies Flk1 signalling and consequently reinforces VEGF-driven responses [39]. In MDD patients, the low VEGF and VEGFR2 resulted in corresponding changes of PIGF.

IGF-1 is produced by different cells in the central and peripheral nervous systems and exerts cell growth, differentiation, maturation and metabolic processes mainly through the IGF-1R. We found no different serum IGF-1 levels in PSD group compared with Non-PSD and MDD patients. This was in line with previous report by Denti et al [40], which displayed that IGF-1 levels were lower in patients with stroke than in controls and further found decreased IGF-1 levels were highly predictive of poor post-stroke outcome at 3 and 6 months, independent of other clinical covariates (e.g. age and stroke severity). Other study proved from the reverse side that the IGF-I accumulation in the site of ischemic injury improved neurological function after ischemia [41]. Besides that, IGF-1 is known to increase the synthesis and activity of other neurotrophic factors (e.g. BDNF, VEGF), which are required to enhance neuronal survival and plasticity in the ischemic brain [42]. Contrary to serum protein, we found higher levels of serum IGF-1 mRNA in PSD patients than in MDD individuals and this represented a statistically significant difference. It was speculated that chronic continuous hyperactivity of the HPA axis in MDD patients might be responsible for this changes. Moreover, increased IGF-1 and IGF-1R was shown to inhibit inflammatory processes, mainly through inhibiting the expression of proinflammatory cytokines, reducing neurodegeneration and secondary low immune function after stroke.

Given the early PSD is difficult to identify, the most appropriate time for predictive analysis for PSD when individuals suffer stroke. Although VEGF and IGF-1 are thought to be important factors in pathophysiology of depression because of their neurotrophic effects [8, 43, 44], unfortunately, no different neurotrophic factor was observed in PSD and Non-PSD group. In hence, we applied this combination with cumulative effect to predictive the onset of PSD, a general predictive performance (AUC = 0.805) was obtained. Intriguingly, when we established the model of the biomarker combination to identify PSD patients from primary depression, we found it could well discriminate between PSD and MDD (AUC = 0.984). It will provide great help for the differential diagnosis in clinical work.

## LIMITATIONS

Our study has several limitations. Firstly, our sample was relatively small, so the applicability was circumscribed. Secondly, we assessed plasma levels of several factors at a single time point to establish a baseline. Thirdly, the age and gender of the four groups were not matched perfectly, even considering possible effects of confounders such as age, gender and education level in statistical analysis.

## CONCLUSIONS

The present study represents the first report evaluating serum and leukocyte VEGF, VEGFR2, PIGF, IGF-1, IGF-1R in PSD patients. The established models of the selected analytes, which can distinguish PSD patients with other depressive type (MDD), potentially reveal new insights into the progressive understood of PSD.

## MATERIALS AND METHODS

### Study population

The present study recruited 39 PSD patients, 42 Non-PSD patients, 40 MDD patients and 38 NC during the period from July 2013 to December 2014. All participants were right handed and gave informed consent to take part in this study, which was approved by the Medical Ethics Committee for Clinical Research of Zhongda Hospital Affiliated to Southeast University. The clinical diagnosis of stroke was performed by neurologist and confirmed through computed tomography (CT) or magnetic resonance imaging (MRI). PSD were satisfied with the following diagnostic criteria: (1) Had stroke before, or stroke occurs earlier than depressive symptoms; (2) Met at least two depressive symptoms except core criterion symptoms of depressed mood and loss of interest or pleasure in nine symptoms of major depressive disorder in DSM-IV; (3) Impairment to fit personal and work functioning; (4) Depressive symptoms lasting more than one week; (5) Free of other major psychiatric disorders, including schizophrenia, bipolar disorder, substance abuse (caffeine, nicotine and alcohol) [45]. The patients meet the diagnostic criteria for MDD using a Structured Clinical Interview according to the DSM-IV by two trained senior psychiatrists. HDRS and MMSE were used to evaluate the serve of depression and cognitive function respectively [46, 47].

### Blood collection

Venous blood samples were drawn from the antecubital veins to EDTA-anticoagulant and coagulant tubes of all participants. Blood samples in anticoagulant was directly stored at −80°C, while in coagulant tubes was centrifuged for 30 min at 3000 rcf, then serum samples were isolated and stored at −80°C until the assay was performed.

### Determination of serum protein level using Enzyme-Linked Immunosorbent Assay (ELISA)

Serum concentrations of VEGF, VEGFR2, PIGF, IGF-1 and IGF-1R were measured using ELISA kits (Human VEGF, VEGFR2, PIGF, IGF-1 Quantikine kit, R&D Systems, MN, USA; ab100546-IGF-1R human ELISA kit, abcam, UK) according to the manufacturer's instructions. The concentrations were expressed as pg of protein/ml except IGF-1 with ng/ml of serum. The detection limit of VEGF, VEGFR2, PIGF, IGF-1 and IGF-1R were 9 pg/ml, 4.6 pg/ml, 7 pg/ml, 0.026 ng/ml and 6 pg/ml respectively.

### Quantitative real-time polymerase chain reaction (qRT-PCR)

Total RNA was extracted from the peripheral blood lymphocytes of whole blood samples using QIAamp RNA Blood Mini Kit (Qiagen, Hilden, Germany) following the manufacturer's protocol. One micrograms of total RNA was used for cDNA reversion by random hexanucleotide primers and Sensiscript Reverse Transcription Kit (Qiagen, Hilden, Germany) according to the instruction manual after assessing RNA quality and quantity with NanoDrop. qRT-PCR were run in triplicate using ViiA7^TM^ sequence detection system (Applied Biosystems, Foster City, CA) by means of incorporation of SybrGreen fluorescent dye and the primer was designed by Primer Express Software v2.0 (see Table 1). qRT-PCR was processed in a final volume of 16ul (1ul cDNA, 8ul 2×SYBGEEN PCR mix, 1ul of each primer and 5ul H_2_O). Amplification commenced with denaturation at 95°C for 2 min followed by 40 cycles at 94°C for 10s, 59°C for 10s and 72°C for 40s. The results were automatically calculated with the data analysis module. Relative expression levels were measured by 2^−ΔΔCT^ methods using glyceraldehyde-3-phosphate dehydrogenase (GAPDH) as endogenous control [48].

### Statistical analysis

Demographic and clinical characteristics were described with mean (M) and standard deviation (SD). The chi-square test was used to evaluate categorical variable, nonparametric test (Kruskal-Wallis H test) and one-way analysis of variance (ANOVA) were used to evaluate continuous variables of general characteristics, clinical and biological changes. The data exceeding M±3SD as outlier were eliminated.

In order to identify the prediction of these neurotrophic factors to PSD and the differentiated function from MDD, discriminant analysis and ROC curve expressed as AUC with the corresponding 95% CI were used.

All analyses were conducted using SPSS Version 20.0 statistical software (SPSS Inc. Chicago, IL).
